# Evaluation of Intracellular Signaling Downstream Chimeric Antigen Receptors

**DOI:** 10.1371/journal.pone.0144787

**Published:** 2015-12-23

**Authors:** Hannah Karlsson, Emma Svensson, Camilla Gigg, Malin Jarvius, Ulla Olsson-Strömberg, Barbara Savoldo, Gianpietro Dotti, Angelica Loskog

**Affiliations:** 1 Department of Immunology, Genetics and Pathology, Science for Life Laboratory, Uppsala University, Uppsala, Sweden; 2 Department of Medical Sciences, Uppsala University, Uppsala, Sweden; 3 Section of Hematology, Uppsala University Hospital, Uppsala, Sweden; 4 Center for Cell and Gene Therapy, Baylor College of Medicine, Houston, Texas, United States of America; University of Oslo, NORWAY

## Abstract

CD19-targeting CAR T cells have shown potency in clinical trials targeting B cell leukemia. Although mainly second generation (2G) CARs carrying CD28 or 4-1BB have been investigated in patients, preclinical studies suggest that third generation (3G) CARs with both CD28 and 4-1BB have enhanced capacity. However, little is known about the intracellular signaling pathways downstream of CARs. In the present work, we have analyzed the signaling capacity post antigen stimulation in both 2G and 3G CARs. 3G CAR T cells expanded better than 2G CAR T cells upon repeated stimulation with IL-2 and autologous B cells. An antigen-driven accumulation of CAR+ cells was evident post antigen stimulation. The cytotoxicity of both 2G and 3G CAR T cells was maintained by repeated stimulation. The phosphorylation status of intracellular signaling proteins post antigen stimulation showed that 3G CAR T cells had a higher activation status than 2G. Several proteins involved in signaling downstream the TCR were activated, as were proteins involved in the cell cycle, cell adhesion and exocytosis. In conclusion, 3G CAR T cells had a higher degree of intracellular signaling activity than 2G CARs which may explain the increased proliferative capacity seen in 3G CAR T cells. The study also indicates that there may be other signaling pathways to consider when designing or evaluating new generations of CARs.

## Introduction

T cells engineered with chimeric antigen receptors (CARs) have shown promising results in patients with hematological malignancies [1–5]. CARs consist of the single chain fragment (scFv) of an antibody fused to a signaling chain e.g. the zeta chain of the TCR/CD3 complex [6]. The first generation (1G) CARs specifically killed target cells and secreted IL-2 upon target recognition *in vitro* [6], but had limited expansion and *in vivo* persistence in the clinic [7–9]. Therefore, a costimulatory endodomain derived from either CD28, 4-1BB or OX40 has been added to the constructs to form a second generation (2G) CAR. Inclusion of CD28 in 2G CARs increased T cell proliferation [10–13], increased cytokine secretion upon target recognition [13–15], promoted CAR T cell persistence to T regulatory cells (Tregs), IL-10 and TGFβ [10] and improved antitumor effect in *in vivo* models [16]. CARs containing 4-1BB showed an increased cytokine secretion, an upregulation of anti-apoptotic genes and enhanced *in vivo* persistence [17–19]. 2G CARs containing 4-1BB have so far shown the most persistent results in patients. In the first report, two out of the three treated chronic lymphocytic leukemia (CLL) patients had complete responses [2]. To date, multiple patients have been treated with the 4-1BB or CD28 2G CAR and impressive effects have been noted in leukemic patients [1–3, 5], and lately also in lymphoma [4]. However, lymphoma patients need critical levels of preconditioning to reach complete response, which may be due to the solid character of these tumors. To further strengthen CARs, third generation (3G) CARs that contain two co-stimulatory elements, for example from both the CD28 and 4-1BB intracellular portions, have been developed [20–26]. The addition of 4-1BB as a second co-stimulatory molecule in the 2G CD28 CAR construct rendered more potent *in vivo* tumor responses [18]. CARs containing 4-1BB or both CD28 and 4-1BB have also showed superior *in vivo* expansion and anti-tumor efficacy compared to CARs carrying CD28 [19, 27]. The persistence of 4-1BB or CD28 2G CAR T cells in patients has been discussed [28] and in clinical trials so far, it appears that time to relapse is longer in patients treated with CARs containing 4-1BB compared to CD28 CARs, indicating an increased persistence of the 4-1BB CAR T cells [5, 29, 30]. Despite increasing knowledge of the therapeutic effect of 2G and 3G CAR T cells, studies of the intracellular signaling downstream CAR is lacking.

In the present study, we compare 2G CAR T cells containing CD28 to a 3G CAR containing both CD28 and 4-1BB to generate a rationale for the use of the latter in clinical trials. We investigated the functional capacity of 3G compared to 2G CARs and have initiated a mapping of the intracellular signaling capacity post antigen stimulation in both 2G and 3G CARs.

## Materials and Methods

### Patient material

PBMCs were isolated from blood of patients with CLL (n = 4) or healthy donors (n = 2) using Ficoll paque gradient centrifugation (Ficoll paque Premium; GE healthcare Life sciences, cat no 17-5442-03). Written consent was obtained from all patients in concordance with the Helsinki Declaration and the study was approved by the Uppsala Regional Ethical Review Board, Uppsala, Sweden (DNr: 2006:145). Peripheral blood from healthy donors was obtained from the blood bank at Uppsala University Hospital. Deidentified cord blood (CB) units were obtained through the MD Anderson Cord Blood Bank (University of Texas, Houston, TX) on a Baylor College of Medicine (BCM) IRB-approved protocol.

### Cell culture

CD19+ Daudi [31] (EBV positive Burkitt’s lymphoma) and CD19- K562 (chronic myeloid leukemia (CML) cell line and NK target) was purchased from ATCC (cat no CCL-213 and CCL-243, respectively) and cultured in RPMI medium (cat no 21875–034) supplemented with 10% fetal bovine serum (cat no 10500–064) and 1% Penicillin-Streptavidin (cat no 15140–122). 293T (ATCC, cat no CRL-3216) was cultured in IMDM medium (cat no 12440–053) supplemented with 10% fetal bovine serum, 1% Penicillin-Streptavidin and 0.1% Sodium Pyruvate (cat no 11360–070). All cell culture components were purchased from Life Technologies.

### Plasmid construction and retrovirus production

The plasmid pRSV-γ [6] contained the original CAR gene consisting of a scFv from an antibody combined with the gamma (γ) chain. To construct a CD19-targeting CAR, the scFv in the pRSV-γ was changed to an anti-CD19 scFv subcloned from the pHBStrep plasmid [32]. The CAR has a hinge domain with the CH2-CH3 motif (long hinge) to link the scFv to the intracellular domain. To increase signaling from the CAR, the γ-chain was changed to the intracellular part of the CD3-zeta (ζ) chain subcloned from the pGEMz plasmid [33]. The CAR construct was then subcloned into the murine Moloney based retroviral backbone plasmid SFG [34]. This step completed the 1G CAR construct that has been used to generate 2G and 3G CAR T cells in the present study. The transmembrane and intracellular region of CD28 was inserted into the SFG after the hinge and prior to the CD3-ζ by recombination to generate 2G CAR T cells [10]. Finally, the intracellular region of 4-1BB was inserted between the CD28 and CD3-z regions to create the 3G CAR construct: SFG.CAR.CD19.28.4-1BBzeta. Virus vectors were produced through Genejuice-mediated (Genejuice; Novagen Inc, cat no 70967) transfection of 293T cells with the vector plasmid together with plasmids containing the gag-pol genes (PegPam; gift of Dr Elio Vanin, Baylor College of Medicin, Houston, TX, USA), and the RD114 envelope (RDF-114; gift of Mary Collins, London, UK). 2G, 3G and Mock (empty vector) virus were made in parallel. Viral supernatants were harvested after 48 and 72 hours, frozen and stored at -80°C.

### Generation of CAR T cells

CAR T cells were generated from peripheral mononuclear blood cells (PBMCs) and cultured in RPMI medium supplemented with 10% fetal bovine serum and 1% Penicillin-Streptavidin, all purchased from Life Technologies. B cells were isolated using αCD20 magnetic beads (CD19+ cells post isolation; mean 96.4% ranging from 90.5 to 99.5%) and T cells were subsequently purified from the CD20 negative fraction using pan T magnetic beads (CD3+ cells post isolation; mean 99.3% ranging from 99 to 99.7%), both from Miltenyi Biotec GmbH (cat no 130-091-104 and 130-096-535, respectively). T cells were activated with 1μg OKT-3/mL (Biolegend, cat no 317315) at day 0. On day 2, T cells were stimulated with 100IU IL-2/mL (Proleukin^®^, Novartis) and the following day (day 3) transduced on RetroNectin-coated (RetroNectin; Takara Bio Inc, cat no T100B) plates pre-incubated with retroviral supernatant and kept in 100IU IL-2/ml.

### T cell culture with repeated antigen stimulation

B cells were isolated from PBMCs through αCD20 magnetic bead isolation. CD20 negative cells were subsequently purified using pan T magnetic beads to generate CAR T cells (Miltenyi Biotec GmbH). Due to the slow proliferative capacity of T cells generated from leukemic patients, the expansion was ongoing for 14 days. On day 14, 1x10^6^ CAR T cells were cultured without stimuli or stimulated with 50IU IL-2/ml twice a week and/or autologous B cells (1:4 ratio, B:T) once a week (S1 Fig) similarly as a previously established protocol to generate EBV specific T cells by stimulating T cells with EBV infected B cells. The cells were harvested and counted once a week during one month’s time. For non-expanding cell cultures, all cells were replated and additions of B cells where calculated based on remaining T cell number in order to maintain the 4:1 T to B cell ratio. In cultures containing more than 1x10^6^ cells, excess cells were used for flow cytometry and 1x10^6^ cells replated. Proteins were extracted from untransduced, Mock, 2G CAR and 3G CAR transduced T cells at day 14 (before co-culture) and 3 days after the last B cell stimulation (endpoint of co-culture) and used for Western blot and cell signaling multiplex assay. Cell culture supernatants were collected 24h after B cell stimulation (once/week), and analyzed for IFNγ (ELI-pair; Diaclone, 851.560.005) and Granzyme B (Diaclone, cat no 850.790.096) using ELISA.

### Flow cytometry

Cells were incubated with 1% BSA in PBS for 10 min, centrifuged and stained for 15 min with the following antibodies: CD3-FITC (cat no 300406), CD4-APC/Cy7 (cat no 300518), CD8-PE/Cy7 (cat no 301012), CD19-PerCP (cat no 302228), CCR7-BV421 (cat no 353208), CD45RA-APC/Cy7 (cat no 304128), CD27-PE (cat no 302808), CD28-PE/Cy7 (cat no 302926), Tim-3-BV421 (cat no 345007), PD-1-PE (cat no 329906) all purchased from Biolegend, α-CAR-DyLight649 AffiniPure F(ab')_2_ Fragment Goat Anti-Human IgG (H+L) purchased from Jackson ImmunoResearch Europe LTD (cat no 109-496-088), or negative isotype control antibodies (Biolegend). Cells were washed with PBS. Analyzed on BD Canto II (BD Biosciences, San Jose, CA) and evaluated with FlowJo (Treestar, Ashland, OR, USA). The transduction efficiency of 3G CAR was lower than that of 2G CAR. Therefore, a correction factor was calculated based on the CAR expression of the 2G and 3G T cells from the same donor at the same time point. This correction factor was then used to normalize the values of the functional assays.

### Kinase assay

B cells were isolated from PBMCs through αCD20 magnetic bead (Miltenyi Biotec GmbH) isolation and the CD20 negative fraction was subsequently purified using pan T magnetic beads all according to manufacturer’s protocol. Pan T purified T cells were used to generate Mock, 2G and 3G CAR T cells. The T cells were stimulated with αCD20-labelled B cells (4:1 ratio) for 1 and 3h. At these time-points the co-culture was run through a LS column (Miltenyi Biotec GmbH) and the T cell fractions (99.9% CD3+) were washed with cold PBS and resuspended in lysis buffer containing M-PER mammalian protein extraction reagent (cat no 78503) supplemented with 1% Halt phosphatase inhibitor cocktail (cat no 78415) and 1% protease inhibitor cocktail (cat no 1861277) all from Thermo Scientific. Protein lysates were assayed for kinase tyrosine phosphorylation analyzed on PamStation12 (PamGene, 's-Hertogenbosch, The Netherlands).

### Cell signaling multiplex assay

The MILLIPLEX® MAP T-Cell Receptor Signaling Magnetic Bead Kit 7-Plex from Millipore Merck (cat no 48-690MAG) was used to detect changes in phosphorylated CREB (Ser133), Erk/MAP kinase 1/2 (Thr185/Tyr187), and phosphorylated tyrosine residues on CD3 epsilon chain, Lck, ZAP-70, LAT, and Syk in T cell lysates from day 14, hence before and 3 days after the last B cell stimulation (endpoint of the co-culture). The assay was set up according to manufacturer’s instructions loading 15μg protein/well and analyzed on the Luminex® system.

### ^51^Chromium-release assay

Target cells (Daudi and K562) were labeled with radioactive chromium (^51^Cr, PerkinElmer, cat no NEZ030S005MC) for 2 hours and subsequently washed prior to co-culture with CAR T cells at different ratios for 4 hours. Co-culture supernatants were mixed with Optiphase supermix (PerkinElmer, cat no 1200–434) and analyzed on the beta counter Wallac Trilux, 1450 microbeta (PerkinElmer, Waltham, MA, USA). Cytotoxicity (% lysis) was calculated as follows: (sample-spontaneous release)/(maximum release–spontaneous release) x 100.

### *In vivo* experiments

C57BL/6J nu/nu mice (Taconic M&B, Ry, Denmark) were injected subcutaneously with 5x10^6^ Daudi cells mixed 1:1 with Matrigel (BD Biosciences, cat no 354248) and treated upon measurable tumor (2 weeks post tumor cell injection) with 5x10^6^ 2G, 3G or mock transduced human T cells via i.p. injection (6 mice per group). Survival and tumor size was monitored throughout the experiment using a caliper. Mice were euthanized if tumor ≥1cm^2^. Animals were housed at the Rudbeck Animal Facility and cared for by the staff according to local regulations. The *in vivo* experiments were approved by Uppsala Animal Ethics Committee (ref no: C319/9).

NSG mice 6 weeks of age (Jackson laboratories) were sublethally irradiated (280Gy) and engrafted with 2x10^5^ CD34^+^ cells selected from umbilical cord blood (UCB) units (from MD Anderson UCB bank, under BCM approved protocol). Engraftment was confirmed within 6 weeks by measuring human CD45^+^ and CD19^+^ cells in the peripheral blood by flow cytometry. Engrafted mice received via tail injection T cells derived from the corresponding UCB negative fraction, transduced with 2G or 3G vector construct and further labeled with FFluc (3x10^6^/mouse). *In vivo* expansion of T cells was monitored over time by IVIS imaging (Xenogen, Caliper Life Sciences Hopkinton, MA), while the elimination of CD19^+^ cell was measured by flow cytometry of peripheral blood samples. By day 60 or 100 mice were euthanized and spleen, bone marrow and blood collected for flow cytometry analysis (human CD45^+^ and CD3^+^). CAR expression by T cells was detected using the Cy-5-conjugated goat anti human IgG (H+L) antibody (Jackson ImmunoResearch) that recognizes the human IgG1-CH2-CH3 component incorporated as a spacer region within the CARs. Samples were analyzed in a FACSCalibur (BD Pharmingen) and were analyzed by CellQuest pro software (BD Biosciences). At least 10000 positive events were acquired in each sample. The NSG mice were maintained at Baylor College of Medicine Animal Facility on a BCM IACUC approved protocol.

### Statistical analysis

Statistical evaluations were performed using GraphPad Prism (GraphPad Software, La Jolla, CA). Differences between groups generating p-values <0.05 were considered significantly different.

## Results

### CAR is expressed on transduced T cells

**Fig 1:**
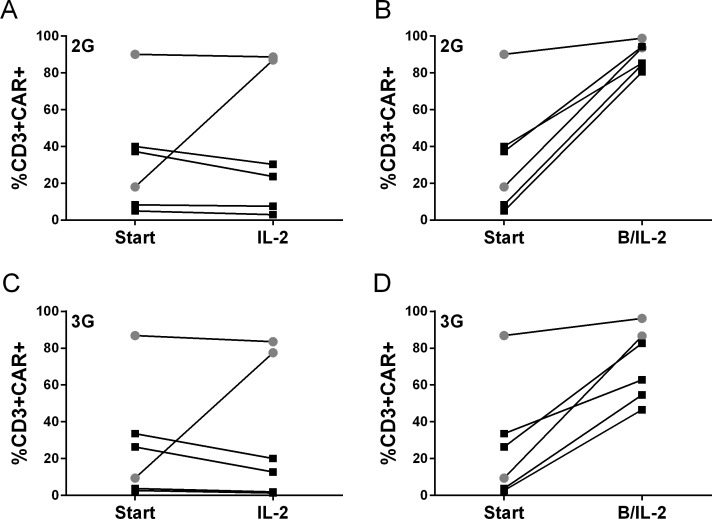
Transduced T cells express CAR. CAR expression on 2G (A, B) and 3G (C, D) T cells was evaluated by flow cytometry before and after co-culture. T cells were gated as viable singlet cells expressing CD3. Statistical differences were calculated using Wilcoxon matched pairs signed rank test (start vs B/IL-2 3G; p = 0.0313, 2G; p = 0.0313). Healthy donors are shown as grey circles and CLL patients as black squares.

Activated T cells from healthy donors and CLL patients were efficiently transduced with Mock, 2G or 3G virus (MLV vector), resulting in a median 2G CAR expression of 33% ranging from 5 to 90% and a 3G median CAR expression of 27.1% ranging from 3 to 87% at day 14,prior to stimulation. There was a difference in CAR expression when comparing 2G and 3G CAR and also between donors. Therefore, to enable comparison of 3G CAR T cells to 2G, CAR expression was normalized for all functional assays. T cells were expanded with autologous B cells, IL-2 or a combination of the two. After one month’s expansion, CAR T cells stimulated with both B cells and IL-2 had a significantly larger fraction of CAR positive cells (Fig 1B and 1D) as well as a higher intensity of expression (data not shown) while CAR T cells cultured with IL-2 alone maintained the same expression level (Fig 1A and 1C). T cells stimulated with B cells alone did not survive during the culture assay since they depended on IL2, hence, no reliable analysis could be performed.

### CAR T cells exhibited a memory-like phenotype and low expression of exhaustion markers

**Fig 2:**
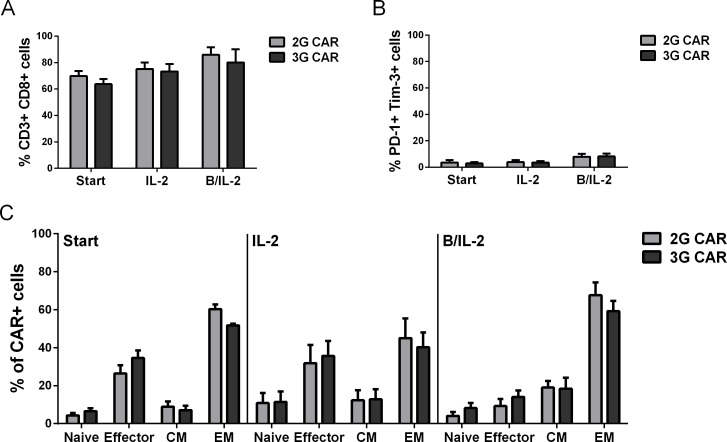
The majority of CAR T cells are CD8+ effector or effector memory cells. Lineage (A; CD3+ CD8+), exhaustion phenotype (B; PD-1+ Tim-3+) and memory/effector status (C) were evaluated by flow cytometry at start and after co-culture with autologous B cells and IL-2 or IL-2 alone. Graphs are showing CAR+ T cells, gated as viable singlet cells expressing CD3 and CAR. For memory/effector status the cells were divided into the following subtypes: naïve: CD45RA+ CCR7+, effector: CD45RA+ CCR7-, central memory (CM): CD45RA- CCR7+ and effector memory (EM): CD45RA- CCR7-. There was no statistical difference in expression of lineage or exhaustion markers between the different time-points or between groups. T cells of effector memory phenotype increased (IL-2 vs B/IL-2 3G: p = 0.0836, 2G: p = 0.1232), while effector cells decreased (start vs B/IL-2 3G: p = 0.0625, 2G: p = 0.0625). There was also a tendency to increased number of central memory cells (start vs B/IL-2 3G: p = 0.0625, 2G: p = 0.0625). Error bars represent SEM. Statistical differences were calculated with unpaired t-test with Welch correction and Wilcoxon matched-pairs signed rank test.

3G CAR T cells were harvested and phenotyped using multicolor flow cytometry before and after one month’s stimulation with IL-2 +/- B cells. As controls, 2G CAR T cells and Mock transduced T cells were used. The majority of cells were of CD8 lineage, yet there were CD4+ T cells present in all cultures (from all donors) (Fig 2A). CAR positive cells showed no difference in CD4/CD8 ratio before or after co-culture, nor was there a difference seen when comparing CAR negative and CAR positive T cells within the cultures (data not shown). The skewing towards CD8+ phenotype was not dependent on transduction but rather dependent on the OKT-3/IL-2 activation. Exhaustion was defined as PD-1+/Tim-3+ double positive cells and were found in less than 16% of the total cell number (Fig 2B). There was no significant difference within the cohorts before and after co-culture. However, CAR- T cells in the 3G CAR culture showed significantly lower number of PD-1+/Tim-3+ cells compared to the CAR+ fraction (data not shown).

An extended T cell phenotype was determined in the different groups before and after expansion. The phenotypes were defined as follows; naïve: CD45RA+ CCR7+, effector: CD45RA+ CCR7-, central memory: CD45RA- CCR7+ and effector memory: CD45RA- CCR7-. CAR or Mock transduced T cells had a similar phenotype prior to co-culture independently of CAR expression. The majority of T cells had a memory-like phenotype of either central or effector type (Fig 2C). After expansion with IL-2 and B cells 3G CAR T cells showed a tendency to increased number of effector memory cells that, however, did not reach significance due to donor variance (3G: p = 0.0836, 2G: p = 0.1232) compared to cells cultured with IL-2 alone. There was also a tendency to increased number of central memory cells compared to before co-culture (3G: p = 0.0625, 2G: p = 0.0625). Cells of effector phenotype showed a tendency to decreased cell number (3G: p = 0.0625, 2G: p = 0.0625) compared to before expansion (Fig 2C). Interestingly, the phenotypically naïve population was maintained despite repeated stimulations with antigen. Hence, the majority of 3G CAR T cells were CD8+ memory cells, expressing low levels of exhaustion markers. The phenotype was also analyzed on the CAR- cells within the 2G and 3G groups. These cells did not change their phenotype post expansion, which was expected since the cells had not been stimulated with antigen (S2 Fig).

### Intracellular signaling downstream CAR T cells

**Fig 3:**
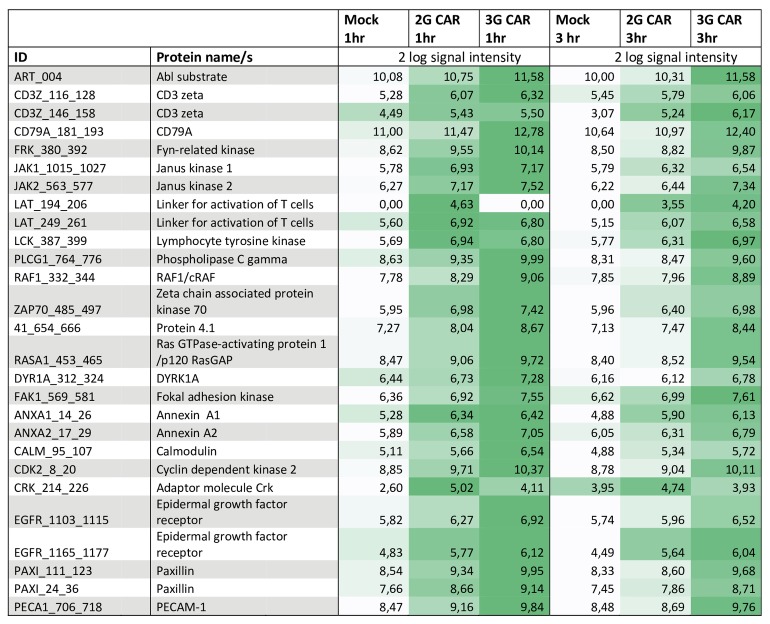
Tyrosine kinase phosphorylation downstream CAR. Tyrosine kinase phosphorylation in mock, 2G or 3G transduced T cells, 1 and 3 hours post stimulation with autologous B cells. Darker color indicates increased signal intensity. Data was normalized for CAR expression. A selection of the most interesting phosphorylation targets in the assay is shown. Complete array data can be found in S1 Table.

To evaluate the activation status of intracellular signaling pathways downstream CAR and signaling pathways activated secondary to CAR stimulation, CAR T cells were stimulated with autologous B cells and analyzed by the PamGene tyrosine kinase array. The Tyrosine Kinase PamChip® Array by PamGene consists of 144 peptides with known phosphorylation sites derived from 100 different proteins. Intact cells or cell lysates can be stimulated and analyzed for the presence of active kinases that will phosphorylate the Array peptides. Hence, an increased phosphorylation of a certain peptide do not mean that the cells contained that certain peptide, but has activated the kinases that can act on those peptides. CAR T cells showed an overall increased activation status compared to Mock transduced cells and 3G T cells were generally more activated than 2G T cells. The activation status in 3G CARs was also maintained over time compared to 2G CARs. Nevertheless, the pattern of what kinases that were activated was similar between 2G and 3G. As expected, signaling molecules downstream of the TCR were phosphorylated also by stimulating CAR receptors such as CD3 zeta, LAT, LCK and ZAP-70. Interestingly, also other proteins involved in regulation of the cell cycle (CDK2), cell adhesion (Paxillin, PECAM-1) and exocytosis (Annexins) were phosphorylated. Stimulating cell adhesion may be of importance especially for treatment of solid tumors, and increased exocytosis may result in enhanced serial killing capacity. The most interesting phosphorylation targets are listed in Fig 3 while the complete array is shown in S1 Table

**Fig 4:**
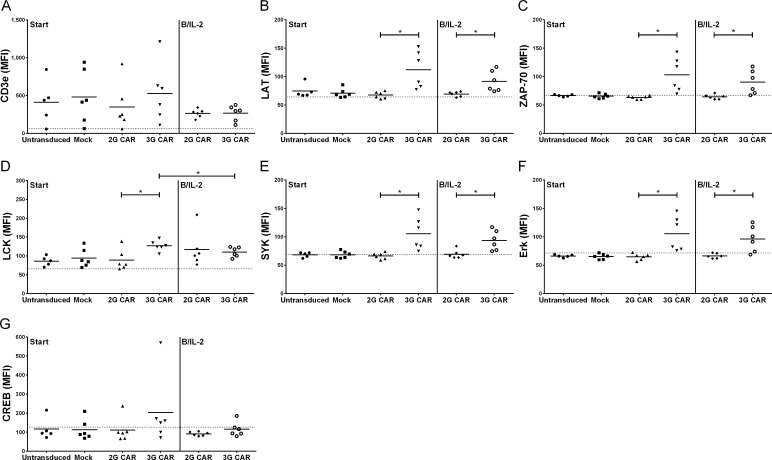
Adaptor proteins involved in TCR signaling are phosphorylated downstream CAR. Phosphorylation of CD3ε (A), LAT (B), ZAP-70 (C), LCK (D), SYK (E), Erk (F) and CREB (G) at start and after stimulation with B cells and IL-2 is demonstrated using MILLIPLEX® MAP T-Cell Receptor Signaling kit. The mean value is indicated in the Figure and the dotted line indicates background levels. Data was normalized for CAR expression. Statistical differences were calculated by unpaired t-test with Welch correction and Wilcoxon matched-pairs signed rank test (* = p<0.05).

Since the PamGene array only demonstrate the capacity of cells to phosphorylate a library of peptides, we confirmed some of the data using the MILLIPLEX® MAP T-Cell Receptor Signaling Magnetic Bead Kit 7-Plex and Western blots. LAT, ZAP-70, SYK and Erk all showed a similar pattern of higher levels of phosphorylated proteins in 3G CAR T cells compared to 2G T cells (Fig 4). Hence, 3G CAR T cells show a different, more activated pattern of intracellular signaling compared to 2G CAR T cells.

### 2G and 3G CARs exhibit equal *in vitro* cytotoxicity

**Fig 5:**
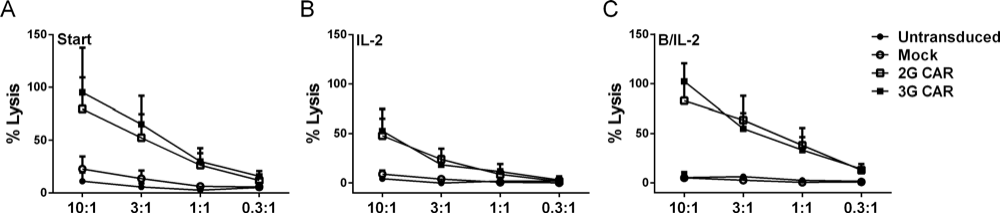
The presence of antigen stimulates and maintains the cytotoxic capacity of CAR T cells. The Figure demonstrates cytotoxicity of CAR T cells generated from CLL patients (n = 3) against CD19+ Daudi before (A) and after co-culture with IL-2 (B) and autologous B cells (C). The cytotoxicity was normalized to CAR expression due to variation of expression. Current CAR expression at each specific time-point was used for normalization. Error bars represent SEM.

Cytotoxic capacity was evaluated with a chromium release assay before and after one month’s expansion. 3G CARs were equally efficient as 2G CAR T cells in specific killing of CD19+ target cells (Fig 5A). The cells also showed some activity against the NK target cell line K562 (data not shown) but this killing was not related to CAR expression as Mock or untransduced T cells showed similar background activity. After co-culture with B cells +/- IL-2, T cells cultured with B cells and IL-2 maintained their cytotoxic capacity (Fig 5C) while T cells cultured with IL-2 alone (Fig 5B) showed decreased cytotoxicity compared to the levels showed prior to co-culture. This effect was most clearly seen with CAR T cells derived from CLL patients.

### Antigen-driven CAR T cell expansion

**Fig 6:**
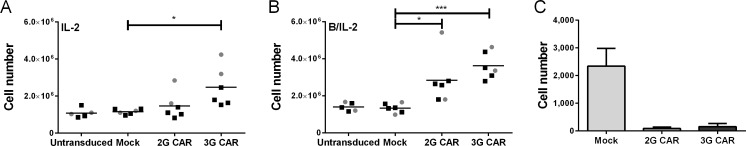
3G CAR T cells show increased proliferation compared to 2G CAR. Proliferation was determined each week through cell count prior to addition of B cells. The Figure shows T cell expansion from start to stimulation with IL-2 (A) or B cells and IL-2 (B). Data was normalized for CAR expression. (C) Remaining viable B cells in co-culture at endpoint (after 2–4 B cell stimulations, 4:1 ratio) analyzed by flow cytometry. The mean is indicated in the Figure. Statistical differences were calculated by unpaired t-test with Welch correction (* = p<0.05, ** = p<0.01, *** = p<0.001).

Proliferation was analyzed weekly during expansion, prior to the addition of autologous B cells and IL-2. After one week’s stimulation, 3G CAR T cells proliferated better than Mock transduced cells (p = 0.0302) (Fig 6A). The proliferation capacity was increased by adding B cells to the IL-2 stimulation (Fig 6B). Both 3G and 2G CAR T cells proliferated more extensively than Mock cells (p = 0.0003 and p = 0.0419, respectively). 3G cells proliferated better than 2G upon antigen stimulation (based on exclusion of the outlier seen in the B/IL-2 2G group, p = 0.0075). CAR T cells efficiently recognized and killed the autologous B cells that were added to the T cell expansions, while they remained in cultures with Mock T cells (Fig 6C).

### CAR T cells respond to antigen with release of IFNγ and Granzyme B

**Fig 7:**
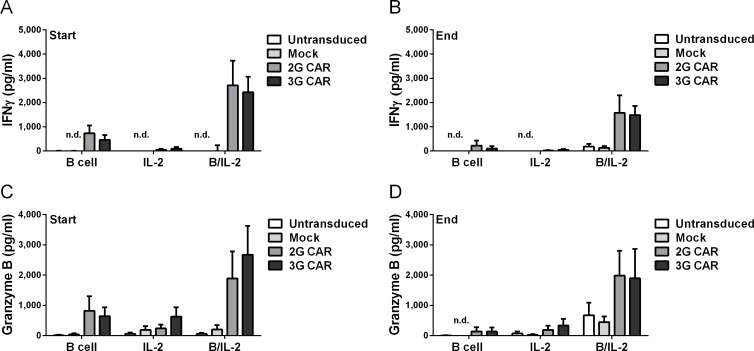
CAR T cells release IFNγ and Granzyme B in response to antigen. Culture supernatants were collected 24h after stimulation at day 1 (start) and at endpoint (end). CAR T cells responded to antigen stimulation through secretion of IFNγ (A, B) and Granzyme B (C, D). IFNγ levels were higher in in the groups stimulated with both B cells and IL-2 compared to B cells alone, both at the beginning (3G: p = 0.0418, 2G: p = 0.1389) and at the end of co-culture (3G: p = 0.0228 p = 2G: p = 0.1446). Data was normalized for CAR expression. Error bars represent SEM. Statistical differences were calculated by unpaired t-test with Welch correction.

Cell supernatants were collected 24 hours post stimulation and analyzed for IFNγ and Granzyme B using ELISA. IFNγ was mainly seen in supernatants from the T cell expansion cultures where the CAR T cells had been stimulated with antigen, while Mock and untransduced cells showed low or undetectable levels (Fig 7A). Granzyme B could be detected in all groups, yet the highest levels were detected in supernatants from CAR T cells cultured with B cells and IL-2 (Fig 7B). IFNγ and Granzyme B levels were higher at first stimulation compared to the levels after repeated stimulations, with the highest levels secreted from CAR T cells stimulated with IL-2 and autologous B cells.

### In vivo tumor cell elimination and expansion of 2G and 3G T cells

**Fig 8:**
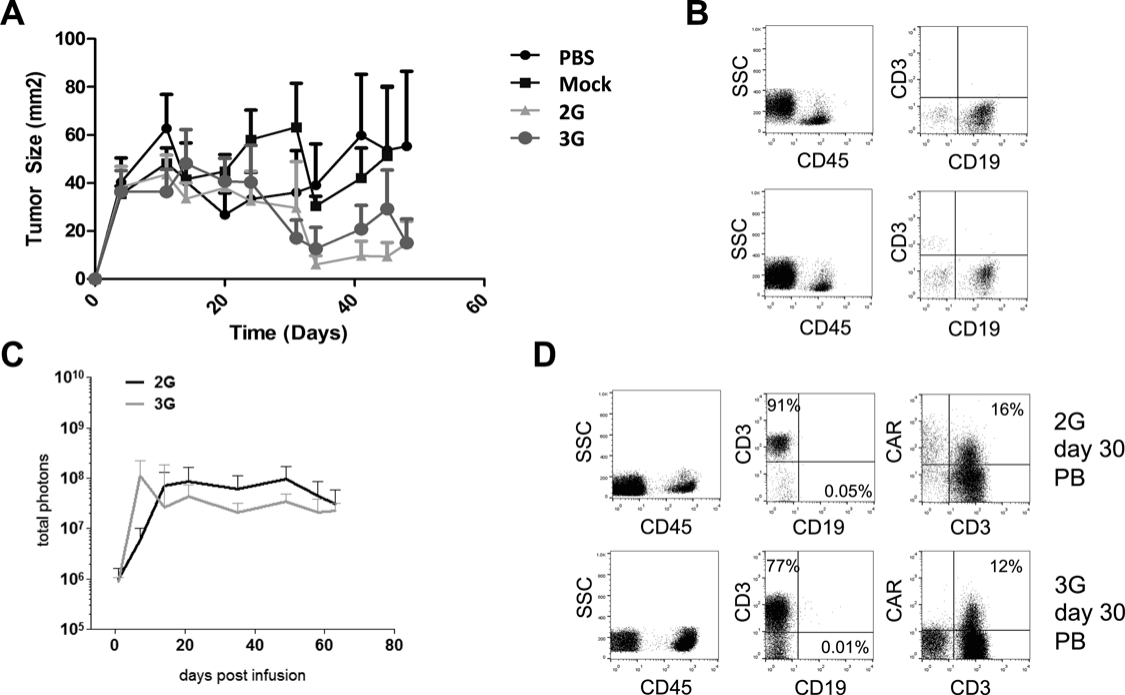
CAR T cells eliminate tumor cells and expand *in vivo*. Mice (Nu/Nu) were injected s.c. with Daudi cells and treated with vehicle, mock, 2G or 3G CAR T cells (6 mice per group). Tumor growth is shown in A. 2G and 3G CAR T cells were infused in NSG mice engrafted with human CD34+ cells (10 mice per group). Successful engraftment is shown as detectable CD45 and CD19 cells in two representative mice (B). Migration and persistence *in vivo* was monitored by GFP-FireFly(FF)-luciferase. The bioluminescence signal from mice infused with 2G (red line) or 3G (black line) T cells is shown in C. At day 30, peripheral blood was collected and the presence of CAR+ T cells and B cells (CD19+) were detected with flow cytometry. T cells and B cells were gated from the CD45+ population. A representative sample is shown in D.

Mice (Nu/Nu) were injected subcutaneously with Daudi cells and mice with established tumors were treated with one 3G or 2G CAR T cell infusion. Both 3G and 2G CAR T cells efficiently reduced tumor growth (Fig 8A). Additionally, humanized NSG mice, engrafted with UBC-derived CD34^+^ cells (Fig 8B) were used to assess the expansion of autologous T cells transduced with 3G or 2G T cells. 2G and 3G CAR T cells expanded equally *in vivo*. Although 3G CAR T cells showed a more rapid initial expansion after infusion no statistically significant differences could be found in the expansion patterns of 2G and 3G T cells (Fig 8C). In addition, both 2G and 3G T cells successfully eliminated circulating CD19^+^ B cells displaying comparable *in vivo* efficacy (Fig 8D). Mice euthanized by day 60 (S3B Fig) or day 100 (S3C Fig) also showed comparable T cell persistence as confirmed by immunophenotype in spleen and bone marrow. No CD19^+^ B cells were detected in mice receiving either 2G or 3G T cells.

## Discussion

The recent success stories of immunotherapy have demonstrated the potency of the immune system to specifically target and kill tumor cells. The induction of Th1 immunity with T cells, M1 macrophages and NK cells as effectors has proven important for potent and sustained responses. *Ex vivo* engineering of the patients’ own T cells with CAR receptors to generate an army of trained killers is now under evaluation for both leukemia, lymphoma as well as for other malignancies [35]. There are multiple options for CAR design and the optimal CAR is yet to be determined. Likely, there are more than one design that will allow for proper T cell activation and persistence. For T cell activation to occur two important signals are required; antigen recognition via binding of peptide-MHC complexes to the T cell receptor (TCR) and costimulation via binding of e.g. CD80/CD86 to CD28 on the T cell. Activation gives rise to upregulation of anti-apoptotic proteins and secretion of cytokines such as IL-2 providing the cells with stimulatory survival signaling [36]. 4-1BB is induced by TCR-signaling. If cross-linked, 4-1BB give rise to increased proliferation and cytokine secretion in the activated T cell [37]. 2G and 3G CARs utilize the costimulatory potential of molecules such as CD28 and 4-1BB to provide the CAR T cells with increased survival signaling. Zhong et al showed higher protein levels of Bcl-xL in antigen-stimulated αPSMA-CAR T cells transduced with a CAR containing both CD28 and 4-1BB. These cells also showed the lowest levels of apoptosis, compared to CAR constructs with only CD28 or 4-1BB or no costimulatory molecules, indicating that the addition of 4-1BB convey an increased resistance to apoptosis [38]. However, there are several other molecules of interest to include alone or in different combinations in CARs, such as OX40 [14, 39, 40] and CD27 [41].

In this study we aimed to evaluate the functional capacity of CD19-targeting 3G CAR T cells with CD28, 4-1BB and CD3 zeta signaling domains and compare their capacity to 2G CARs lacking 4-1BB as well as to Mock transduced T cells. CAR T cells could be generated from both healthy donors and CLL patients but the CAR expression of 2G CARs was always slightly higher than 3G CAR expression both related to number of positive cells and number of CAR molecules per cell as judged by a higher mean fluorescent intensity upon analysis of CAR expression. Hence, for some of the functional assays we correlated the data to the number of CAR+ cells to be able to more appropriately compare the ability of the 2G and 3G cells. Further, the T cells were analyzed before and after repeated stimulation with autologous B cells to determine the persistence of CAR T cells.

Co-culture of CAR T cells with autologous B cells stimulated an accumulation of both 3G and 2G CAR positive cells in the cultures, which was not seen in cultures with IL-2 alone. Further, antigen-driven proliferation generated a memory-like phenotype of the cultured CAR T cells with low expression of the exhaustion markers PD-1 and Tim-3. There is emerging evidence that such a phenotype will generate a better *in vivo* response. For example, in a primate model genetically modified T cells derived of central memory phenotype was shown to persist longer than effector cells derived from effector memory cells [42]. Further, Kalos et al reported that the persisting cells in their CLL patients treated with 2G 4-1BB CAR T cells were of central memory phenotype [2]. A major concern of using more differentiated cells is the risk of transferring exhausted cells. In our CAR T cells cultures, CAR+ cells with a naïve phenotype are also persisting independently of repeated antigen stimulation. It has been suggested that CAR T cells with a more naïve phenotype may be preferred to effector T cells to increase survival and generation of memory subtypes. In a murine model, Hinrich et al showed that effector cells derived from naïve cells displayed superior antitumor activity upon adoptive transfer compared to central memory T cells [43]. Further, Gattinoni et al reported that stem-like T memory cells (CD8+, CD45RA+, CCR7+, CD62L+, CD27+, CD28+) phenotypically resembled naïve cells but carried traits of memory cells [44]. Xu et al noted that the frequency of CD8+, CD45RA+, CCR7+ cells within the infused product correlated with the *in vivo* expansion of CD19-targeting CAR T cells in lymphoma patients [45]. In our study, the antigen-expanded T cells were mainly of memory phenotype while maintaining a small population of phenotypically naïve cells and low levels of exhaustion markers. Hence, antigen-driven expansion of CAR T cells may be an interesting approach for GMP production prior to clinical use. However, 3G and 2G CAR T cells did not differ in regard to phenotype upon repeated antigen stimulation. The cytotoxic capacity of 2G and 3G αCD19-CAR T cells was also similar pre and post prolonged culture with IL-2 or with the addition of autologous B cells. However, the cytotoxic capacity of both 2G and 3G CARs was lower after expansion with only IL-2 suggesting that antigen-driven expansion maintains potent T cells rather than induces exhaustion. Artificial APCs can also efficiently expand CAR+ T cells [46] and can become an important tool when tumor material is scarce and for tumors where expression of co-stimulatory molecules is low or lacking.

The proliferative capacity of 3G CAR T cells upon IL-2 stimulation was higher than that of 2G CAR T cells. 2G CARs were not significantly different from Mock or untransduced cells. Upon repeated stimulation with autologous B cells, both 2G and 3G CAR T cells showed increased proliferation capacity compared to Mock and untransduced cells. With the exception of one donor, 3G CARs had still a higher proliferative capacity than 2G CARs. Barrett et al emphasizes the importance of maintaining proliferation capacity during the *ex vivo* expansion phase of manufacturing, suggesting in the light of recent studies that proliferation capacity might be more important than cytotoxic capacity [47]. Yet, distinct differences in cytotoxicity and persistence between the different types of CAR T cells will most likely be revealed *in vivo* since the complex network of stimulatory and inhibitory pathways are difficult to mimic in preclinical settings. Tammana et al showed that CARs with costimulatory molecule 4-1BB alone or combined with CD28 provided a more robust antitumor response *in vivo* compared to CARs with CD28 alone or without costimulatory molecules [18]. Zhong et al demonstrated an increased survival of mice treated with a 3G CAR (CD28, 4-1BB) compared to either 2G (CD28 or 4-1BB) or 1G CAR T cells [38]. 2G and 3G CAR T cells efficiently reduced tumor volume in a subcutaneous tumor model and eliminated human CD19+ cells in NSG mice. Previous data has suggested elimination of CAR T cells containing a long CH2-CH3 spacer in NSG mice [48], yet CAR T cells in our studies could still be detected at day 100 in NSG mice, also the percentage of CAR+ cells detected was larger for 3G than 2G.

The intracellular signaling events downstream different CARs affect the efficiency and longevity of the T cells. Considering the use of the CD3 zeta domain in the CAR, TCR-associated proteins will likely participate in CAR signaling but it is not known to what extent other signaling pathways are affected in different types of CAR T cells. In this study, the PamGene station was utilized to investigate phosphorylation status of multiple targets. Upon antigen stimulation both 3G and 2G T cells could rapidly activate kinases that have the capacity to phosphorylate a number of proteins. 3G T cells showed a higher activation status than 2G but both were increased compared to Mock T cells. After 3 hours the activation status declined but more slowly in 3G T cells. As expected, all the common proteins downstream the TCR signaling pathway were activated such as CD3 zeta, LAT, LCK and ZAP-70. These data were confirmed also by analyzing the T cells using the MILLIPLEX® MAP T-Cell Receptor Signaling kit. 3G CAR T cells had a significantly higher phosphorylation status on antigen-mediated signaling molecules compared to 2G CAR T cells. The PamGene array also revealed other possibly phosphorylated molecules that will need further confirmation. For example, Calmodulin phosphorylation was increased post antigen stimulation. Calmodulin is a calcium-binding messenger protein important for many Ca2+ regulated pathways in lymphocytes and other cells. For example, upon Ca2+ release from the endoplasmatic reticulum (ER), calcium-bound Calmodulin activates the phosphatase calcineurin, which in turn promotes NFAT-mediated IL-2 transcription [49]. Further, both Annexin A1 and A2 phosphorylation was increased in 2G and 3G CAR T cells. Annexins are a family of Ca2+ dependent phospholipid-binding proteins important in various cellular processes, such as those regulating changes in cell shape and organization of vesicles and exocytosis [50]. They are also involved in inflammation and apoptosis [51]. Annexin A1 promotes T cell activation and seems important to fine-tune TCR signaling. However, it has an inhibitory effect on innate immune cells such as neutrophils [52] and inhibits LPS-mediated DC activation [53], Annexin A2 is phosphorylated upon T cell activation and may be involved in signal transduction during cellular proliferation and differentiation [54].

CDK2 is a protein involved in the progression of the cell cycle as CDK binding to cyclin E during G1 phase promotes the transition from G1 to S phase. Antigen stimulation together with costimulation activates a CDK cascade including CDK2. However, in the absence of costimulation the T cells are entering a state of anergy, which is enforced by the CDK inhibitory protein p27kip1. The major target of p27kip1 is CDK2. In a study of allograft rejection it was shown that CDK2 was highly active in T cells that infiltrated the allograft [55]. Interestingly, cyclin E and CDK2 can in a collaborative effort phosphorylate FoxP3, which reduces FoxP3 stability [56] and may explain the transient FoxP3 expression in recently activated T cells. Correspondingly, it was recently shown that CDK2 inhibition promotes induction of T regulatory cells by enhancing the TGFβ-mediated Smad3 signaling pathway [57]. Hence, the strong and sustained phosphorylation of CDK2 in 3G T cells may be an important factor contributing to the enhanced proliferation compared to 2G T cells.

There were also enhanced phosphorylation of molecules that can potentially be involved in self-regulation of the antigen-induced activation such as Crk, Protein 4.1R, FAK and DYRK1A. Crk adaptor molecules interact with a number of signaling molecules in the cell. Their function is not fully understood but there is one report stating that T cells lacking both Crk and CrkL have a defective chemotactic response to multiple chemokines and may show defective diapedesis. In a cutaneous DTH model, Crk and CrkL were required for efficient T cell migration to sites of inflammation. Crk proteins also controlled T cell activation [58]. Nevertheless, another report demonstrates that Crk is cleaved in response to ER stress to exert a pro-apoptotic function. Crk -/- cells were strongly resistant to ER stress-induced apoptosis [59]. Our data show that Crk activation was somewhat higher in 2G CAR T cells than in 3G CARs. Protein 4.1R binds to LAT and inhibits its phosphorylation of ZAP70 thereby negatively regulating TCR-mediated signaling [60]. FAK instead inhibits TCR signaling by phosphorylation of Lck [61]. If present in T cells, DYRK1A may regulate T cell activation by inhibition of NFAT activation since it has been shown that DYRK1A is involved in NFAT activation in other cells such as cardiomyocytes [62] and in the Drosophila model [63].

Despite of CAR T cell expansion, there was a remaining CAR+ naïve T cell population in the cultures. Ras is activated by TCR stimulation and has a role in activating the MAP kinases leading towards cell growth. Ras is inactivated by Ras GTPase-activating proteins (RasGAPs) such as RASA1. In a paper by Lapinski PE et al it was shown that RASA1 is an important regulator of thymocyte survival during positive selection as well as promoting the survival of naïve T cells in the periphery [64]. In RAFA1 deficient mice, the total number of naïve CD4 and CD8 T cells was reduced by 50–90% and it was shown that RASA1 deficient mice had an impaired IL-7-mediated survival of naïve T cells. There was phosphorylation of RASA1 peptides in the PamGene array indicating that there may be an active process ongoing to maintain a phenotype of naïve cells during antigen-driven expansion. However, this assumption needs to be experimentally verified.

Other proteins that can be phosphorylated in CAR T cells post activation are Paxillin, PECAM-1 and EGFR. Paxillin is a cytoskeletal adaptor protein that localizes to the microtubule-organizing center in T cells and is part of the immunological synapse of cytotoxic T cells [65]. Paxillin is phosphorylated downstream of ERK, hence, connecting it to the TCR-mediated signaling. Another molecule involved in cell adhesion is PECAM-1. Little is known about PECAM-1 in T cells but in a recent paper Proust R et al describes that PECAM-1 may be involved in cell adhesion in T cells [66]. Both Paxillin and PECAM-1 was highly phosphorylated by 3G CAR T cells and the phosphorylation was sustained also 3 hours post antigen stimulation. Finally, phosphorylation of EGFR was seen post antigen stimulation in the CAR T cells. EGFR is ubiquitously expressed but has been regarded absent from hematopoietic cells. However, EGFRs have been demonstrated in some immune cell populations and it has been shown that T regulatory cells express EGFR during inflammatory conditions. In PBMCs, CD4 T cells had low expression levels while it was absent on CD8 T cells [67]. However, it is not known if the EGFR is present on activated T cells. Nevertheless, the EGFR and the TCR share a common signaling pathway via the ERK-MAP kinase molecule and EGFR signaling in T cells could possibly enhance their survival and activation status. Our data suggests that the EGFRs can be efficiently phosphorylated by activated T cells if they are indeed present and 3G T cells can sustain the activation also 3 hours post antigen stimulation.

Taken together, these data demonstrates that antigen-driven expansion of CAR T cells generates memory-like T cells with maintained cytotoxic capacity. Further, while the phenotype and cytotoxic capacity post repeated antigen stimulation were similar between 3G and its predecessor (2G). The 2G and 3G CAR T cells also showed efficient killing *in vivo*. 3G CAR T cells had an increased activation of intracellular signaling pathways concerning both TCR signaling and adhesion molecules compared to 2G CARs. However, the tyrosine kinase array indicates potentially interesting pathways that can be activated or blocked to generate even more efficient CAR designs.

## Supporting Information

S1 Fig Experimental outline of co-culture experiments and overview of cell preparation. A. Experimental outline of the co-culture experiment. 3G, 2G CAR T, mock or untransduced T cells were cultured with autologous B cells, IL-2 or the combination of the two. Proliferation was assessed every week. CAR T cell phenotype and cytotoxic capacity was analyzed before and after co-culture using flow cytometry and chromium release assay, respectively. B. Overview of cell preparation. Autologous T and B cells were isolated from healthy donors and CLL patients using MACS beads. The purity of the B cell population (αCD20-isolation) was confirmed with αCD19 FACS staining (mean expression was 96.4% ranging from 90.5 to 99.5). T cells were isolated from the CD20- fraction using pan T beads, rendering an unlabeled CD3+ population with a mean CD3 expression of 99.3% (range 99–99.7).
(TIF)

S2 Fig Expression levels of memory markers in the CAR negative populations. CAR- T cells were gated as viable singlet CD3+ CAR- cells. Memory phenotype defined as; naïve: CD45RA+, CCR7+, effector: CD45RA+, CCR7-, central memory (CM): CD45RA-, CCR7+, effector memory (EM): CD45RA-, CCR7. CAR negative T cells show similar expression pattern of memory markers as CAR positive cells before co-culture. However, after co-culture with antigen, the increase in memory phenotype and decrease in effector cells was only seen in the CAR positive population.
(TIF)

S3 Fig Persistence of CAR T cells *in vivo*. Comparable expression levels of FF-luciferase was seen in 2G and 3G CAR+ T cells as measured by GFP expression (A). NSG Mice were euthanized on day 60 (B) or day 100 (C). CD19+ B cells were completely eliminated and longterm persistence of CAR+ T cells was seen. T and B cells are gated from the CD45+ population. Panels show phenotype from a representative mouse for each construct.
(TIF)

S1 Table Activation of tyrosine kinases in CAR T cells. The table demonstrates mean values from the tyrosine kinase array by PamGene. CAR T cells (2G and 3G) as well as Mock T cells were stimulated with autologous B cells for 1 and 3 hours. The cells were purified using MACS beads and lysed prior to analysis. Data was normalized for CAR expression.
(DOCX)
