# Supplementary figures and images for: Evaluation of Intracellular Signaling Downstream Chimeric Antigen Receptors
Source: PLoS One. 2015 Dec 23;10(12):e0144787. doi: 10.1371/journal.pone.0144787 (PMC4689545; doi:10.1371/journal.pone.0144787)

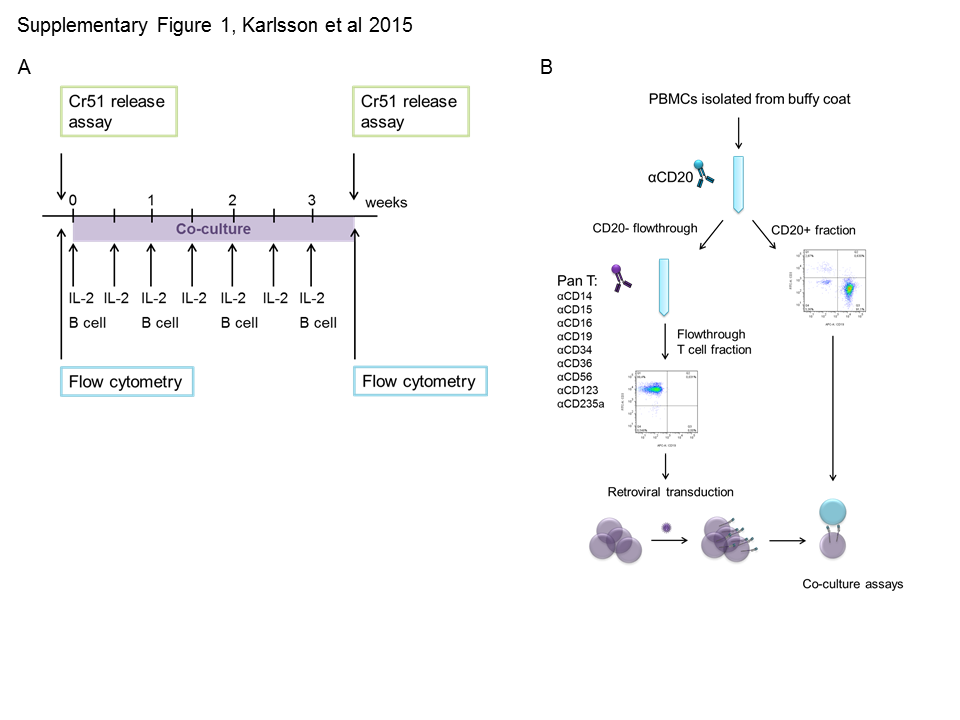

Supplement: S1 Fig — A. Experimental outline of the co-culture experiment. 3G, 2G CAR T, mock or untransduced T cells were cultured with autologous B cells, IL-2 or the combination of the two. Proliferation was assessed every week. CAR T cell phenotype and cytotoxic capacity was analyzed before and after co-culture using flow cytometry and chromium release assay, respectively. B. Overview of cell preparation. Autologous T and B cells were isolated from healthy donors and CLL patients using MACS beads. The purity of the B cell population (αCD20-isolation) was confirmed with αCD19 FACS staining (mean expression was 96.4% ranging from 90.5 to 99.5). T cells were isolated from the CD20- fraction using pan T beads, rendering an unlabeled CD3+ population with a mean CD3 expression of 99.3% (range 99–99.7). (TIF) [file pone.0144787.s001.tif]

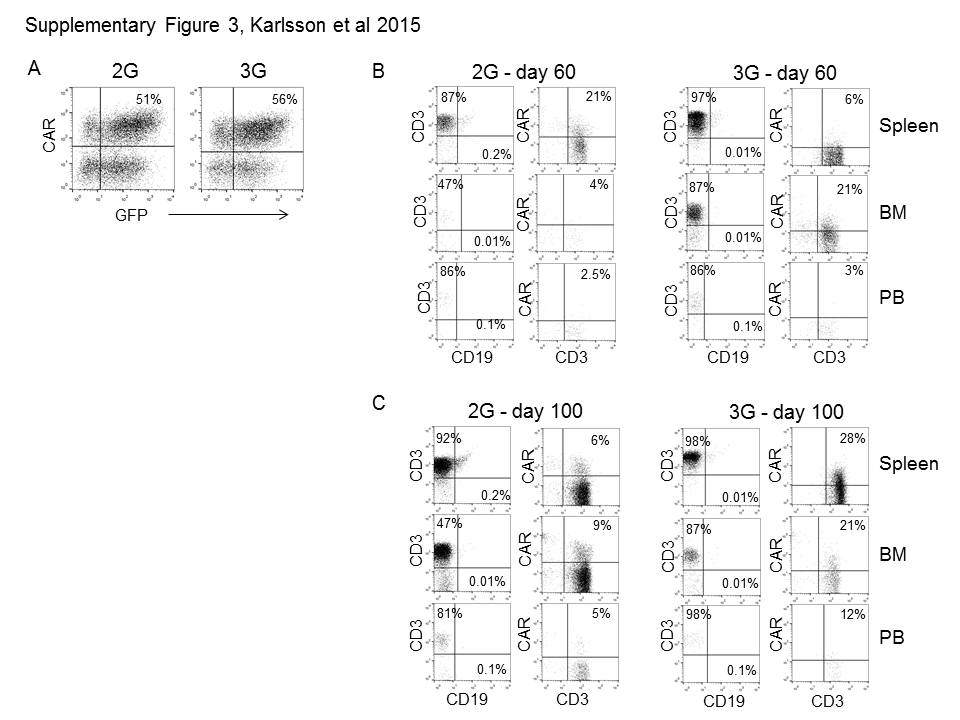

Supplement: S3 Fig — Comparable expression levels of FF-luciferase was seen in 2G and 3G CAR+ T cells as measured by GFP expression (A). NSG Mice were euthanized on day 60 (B) or day 100 (C). CD19+ B cells were completely eliminated and longterm persistence of CAR+ T cells was seen. T and B cells are gated from the CD45+ population. Panels show phenotype from a representative mouse for each construct. (TIF) [file pone.0144787.s003.tif]
