# Supplementary material for: Evaluation of Intracellular Signaling Downstream Chimeric Antigen Receptors
Source: PLoS One. 2015 Dec 23;10(12):e0144787. doi: 10.1371/journal.pone.0144787 (PMC4689545; doi:10.1371/journal.pone.0144787)
Supplement: S1 Table — The table demonstrates mean values from the tyrosine kinase array by PamGene. CAR T cells (2G and 3G) as well as Mock T cells were stimulated with autologous B cells for 1 and 3 hours. The cells were purified using MACS beads and lysed prior to analysis. Data was normalized for CAR expression. (DOCX) [file pone.0144787.s004.docx]

Supplementary Table, Karlsson et al 2015

|  |  |  |  |  | **Mock 1 hr** | **2CAR 1 hr** | **3CAR 1hr** | **Mock 3 hr** | **2CAR 3 hr** | **3CAR 3hr** |
| --- | --- | --- | --- | --- | --- | --- | --- | --- | --- | --- |
| **ID** |  | **Uniprot** | **Sequence** |  | 2 log signal intensity | | | 2 log signal intensity | | |
| 41_654_666 |  | P11171 | LDGENIYIRHSNL |  | 7,27 | 8,04 | 8,67 | 7,13 | 7,47 | 8,44 |
| ACHD_383_395 |  | Q07001 | YISKAEEYFLLKS |  | 4,71 | 5,67 | 5,95 | 4,61 | 4,74 | 5,78 |
| ANXA1_14_26 |  | P04083 | IENEEQEYVQTVK |  | 5,28 | 6,34 | 6,42 | 4,88 | 5,90 | 6,13 |
| ANXA2_17_29 |  | P07355 | HSTPPSAYGSVKA |  | 5,89 | 6,58 | 7,05 | 6,05 | 6,31 | 6,79 |
| ART_004 |  | Na | EAIYAAPFAKKK |  | 10,08 | 10,75 | 11,58 | 10,00 | 10,31 | 11,58 |
| B3AT_39_51 |  | P02730 | TEATATDYHTTSH |  | 0,00 | 5,13 | 5,46 | 3,33 | 4,28 | 5,05 |
| C1R_199_211 |  | P00736 | TEASGYISSLEYP |  | 4,23 | 4,79 | 5,12 | 4,63 | 4,12 | 3,00 |
| CALM_95_107 |  | P62158 | KDGNGYISAAELR |  | 5,11 | 5,66 | 6,54 | 4,88 | 5,34 | 5,72 |
| CBL_693_705 |  | P22681 | EGEEDTEYMTPSS |  | 0,00 | 5,63 | 5,21 | 4,28 | 4,42 | 5,23 |
| CD3Z_116_128 |  | P20963 | KDKMAEAYSEIGM |  | 5,28 | 6,07 | 6,32 | 5,45 | 5,79 | 6,06 |
| CD3Z_146_158 |  | P20963 | STATKDTYDALHM |  | 4,49 | 5,43 | 5,50 | 3,07 | 5,24 | 6,17 |
| CD79A_181_193 |  | P11912 | EYEDENLYEGLNL |  | 11,00 | 11,47 | 12,78 | 10,64 | 10,97 | 12,40 |
| CDK2_8_20 |  | P24941 | EKIGEGTYGVVYK |  | 8,85 | 9,71 | 10,37 | 8,78 | 9,04 | 10,11 |
| CRK_214_226 |  | P46108 | GPPEPGPYAQPSV |  | 2,60 | 5,02 | 4,11 | 3,95 | 4,74 | 3,93 |
| CTNB1_79_91 |  | P35222 | VADIDGQYAMTRA | | 5,60 | 6,35 | 3,93 | 5,47 | 5,77 | 6,18 |
| DCX_109_121 |  | O43602 | GIVYAVSSDRFRS |  | 6,83 | 7,30 | 7,90 | 6,48 | 6,60 | 7,51 |
| DYR1A_312_324 |  | Q13627 | CQLGQRIYQYIQS |  | 6,44 | 6,73 | 7,28 | 6,16 | 6,12 | 6,78 |
| EFS_246_258 |  | O43281 | GGTDEGIYDVPLL |  | 9,91 | 10,78 | 11,57 | 9,42 | 9,74 | 10,85 |
| EGFR_1103_1115 |  | P00533 | GSVQNPVYHNQPL |  | 5,82 | 6,27 | 6,92 | 5,74 | 5,96 | 6,52 |
| EGFR_1118_1130 |  | P00533 | APSRDPHYQDPHS |  | 5,47 | 5,62 | 6,18 | 5,02 | 5,23 | 5,94 |
| EGFR_1165_1177 |  | P00533 | ISLDNPDYQQDFF |  | 4,83 | 5,77 | 6,12 | 4,49 | 5,64 | 6,04 |
| EGFR_1190_1202 |  | P00533 | STAENAEYLRVAP |  | 4,61 | 5,80 | 6,00 | 3,85 | 5,09 | 5,48 |
| ENOG_37_49 |  | P09104 | SGASTGIYEALEL |  | 9,88 | 10,73 | 11,50 | 9,63 | 9,94 | 11,17 |
| EPHA1_774_786 |  | P21709 | LDDFDGTYETQGG |  | 6,66 | 7,61 | 8,00 | 6,19 | 6,92 | 7,66 |
| EPHA2_765_777 |  | P29317 | EDDPEATYTTSGG |  | 6,90 | 7,87 | 8,16 | 6,64 | 7,10 | 7,97 |
| EPHA4_589_601 |  | P54764 | LNQGVRTYVDPFT |  | 5,67 | 5,99 | 6,47 | 5,42 | 5,70 | 5,96 |
| EPHA7_607_619 |  | Q15375 | TYIDPETYEDPNR |  | 6,52 | 7,37 | 7,74 | 6,54 | 6,65 | 7,51 |
| EPHB1_771_783 |  | P54762 | DDTSDPTYTSSLG |  | 5,97 | 6,85 | 6,89 | 5,58 | 6,14 | 6,87 |
| EPHB4_583_595 |  | P54760 | IGHGTKVYIDPFT |  | 5,11 | 5,63 | 6,09 | 5,06 | 5,17 | 5,50 |
| EPOR_361_373 |  | P19235 | SEHAQDTYLVLDK |  | 6,67 | 7,67 | 8,10 | 6,39 | 7,03 | 7,85 |
| EPOR_419_431 |  | P19235 | ASAASFEYTILDP |  | 5,71 | 6,77 | 6,98 | 5,54 | 6,14 | 6,78 |
| ERBB2_1241_1253 |  | P04626 | PTAENPEYLGLDV |  | 0,00 | 5,30 | 4,45 | 3,01 | 4,81 | 5,33 |
| ERBB2_870_882 |  | P04626 | LDIDETEYHADGG |  | 0,00 | 3,63 | 4,59 | 0,00 | 4,12 | 4,68 |
| ERBB4_1277_1289 |  | Q15303 | IVAENPEYLSEFS |  | 3,43 | 4,61 | 5,33 | 4,07 | 4,72 | 5,65 |
| FAK1_569_581 |  | Q05397 | RYMEDSTYYKASK |  | 6,36 | 6,92 | 7,55 | 6,62 | 6,99 | 7,61 |
| FAK2_572_584 |  | Q14289 | RYIEDEDYYKASV |  | 6,98 | 7,87 | 8,33 | 7,05 | 7,22 | 7,93 |
| FER_707_719 |  | P16591 | RQEDGGVYSSSGL |  | 7,23 | 7,91 | 8,35 | 6,92 | 7,19 | 8,02 |
| FES_706_718 |  | P07332 | REEADGVYAASGG |  | 7,49 | 8,42 | 8,91 | 7,31 | 7,64 | 8,63 |
| FGFR1_761_773 |  | P11362 | TSNQEYLDLSMPL |  | 4,33 | 5,83 | 5,96 | 3,98 | 5,31 | 5,12 |
| FGFR2_762_774 |  | P21802 | TLTTNEEYLDLSQ |  | 4,99 | 6,16 | 6,30 | 5,51 | 5,95 | 6,43 |
| FGFR3_753_765 |  | P22607 | TVTSTDEYLDLSA |  | 3,78 | 5,60 | 5,50 | 4,30 | 5,42 | 6,07 |
| FRK_380_392 |  | P42685 | KVDNEDIYESRHE |  | 8,62 | 9,55 | 10,14 | 8,50 | 8,82 | 9,87 |
| INSR_1348_1360 |  | P06213 | SLGFKRSYEEHIP |  | 5,76 | 5,75 | 6,28 | 5,78 | 5,82 | 6,49 |
| INSR_992_1004 |  | P06213 | YASSNPEYLSASD |  | 0,12 | 4,51 | 5,46 | 3,82 | 4,53 | 5,16 |
| JAK1_1015_1027 |  | P23458 | AIETDKEYYTVKD |  | 5,78 | 6,93 | 7,17 | 5,79 | 6,32 | 6,54 |
| JAK2_563_577 |  | O60674 | VRREVGDYGQLHETE | | 6,27 | 7,17 | 7,52 | 6,22 | 6,44 | 7,34 |
| K2C6B_53_65 |  | P04259 | GAGFGSRSLYGLG |  | 7,05 | 7,53 | 8,22 | 6,97 | 6,99 | 7,66 |
| K2C8_425_437 |  | P05787 | SAYGGLTSPGLSY |  | 5,87 | 6,23 | 6,71 | 5,82 | 6,13 | 6,51 |
| KSYK_518_530 |  | P43405 | ALRADENYYKAQT |  | 5,55 | 6,26 | 6,62 | 5,40 | 5,94 | 6,56 |
| LAT_194_206 |  | O43561 | MESIDDYVNVPES |  | 0,00 | 4,63 | 0,00 | 0,00 | 3,55 | 4,20 |
| LAT_249_261 |  | O43561 | EEGAPDYENLQEL |  | 5,60 | 6,92 | 6,80 | 5,15 | 6,07 | 6,58 |
| LCK_387_399 |  | P06239 | RLIEDNEYTAREG |  | 5,69 | 6,94 | 6,80 | 5,77 | 6,31 | 6,97 |
| MBP_198_210 |  | P02686 | ARTAHYGSLPQKS |  | 6,71 | 7,08 | 7,56 | 6,64 | 6,84 | 7,53 |
| MBP_259_271 |  | P02686 | FGYGGRASDYKSA |  | 5,97 | 6,08 | 6,55 | 6,10 | 6,15 | 9,96 |
| MET_1227_1239 |  | P08581 | RDMYDKEYYSVHN |  | 6,62 | 7,42 | 7,86 | 6,54 | 7,03 | 7,62 |
| MK01_180_192 |  | P28482 | HTGFLTEYVATRW |  | 5,72 | 6,26 | 6,72 | 5,31 | 5,52 | 5,79 |
| MK07_211_223 |  | Q13164 | AEHQYFMTEYVAT |  | 6,88 | 7,38 | 8,01 | 7,07 | 7,27 | 8,04 |
| MK12_178_190 |  | P53778 | ADSEMTGYVVTRW | | 5,82 | 6,36 | 6,71 | 5,37 | 5,88 | 6,36 |
| NTRK1_489_501 |  | P04629 | HIIENPQYFSDAC |  | 4,85 | 5,34 | 5,07 | 4,74 | 5,20 | 5,96 |
| NTRK2_696_708 |  | Q16620 | GMSRDVYSTDYYR |  | 7,20 | 7,67 | 8,16 | 7,23 | 7,39 | 8,21 |
| P85A_600_612 |  | P27986 | NENTEDQYSLVED |  | 6,67 | 7,60 | 8,08 | 6,57 | 7,00 | 7,81 |
| PAXI_111_123 |  | P49023 | VGEEEHVYSFPNK |  | 8,54 | 9,34 | 9,95 | 8,33 | 8,60 | 9,68 |
| PAXI_24_36 |  | P49023 | FLSEETPYSYPTG |  | 7,66 | 8,66 | 9,14 | 7,45 | 7,86 | 8,71 |
| PDPK1_2_14 |  | O15530 | ARTTSQLYDAVPI |  | 8,04 | 8,58 | 9,17 | 7,96 | 8,02 | 8,99 |
| PDPK1_369_381 |  | O15530 | DEDCYGNYDNLLS |  | 6,49 | 7,13 | 7,46 | 6,25 | 6,71 | 7,51 |
| PECA1_706_718 |  | P16284 | KKDTETVYSEVRK |  | 8,47 | 9,16 | 9,84 | 8,48 | 8,69 | 9,76 |
| PGFRB_1002_1014 | | P09619 | LDTSSVLYTAVQP |  | 5,81 | 6,72 | 6,80 | 5,49 | 6,12 | 6,80 |
| PGFRB_1014_1028 | | P09619 | PNEGDNDYIIPLPDP | | 4,07 | 6,44 | 6,17 | 4,18 | 5,77 | 6,00 |
| PGFRB_572_584 |  | P09619 | VSSDGHEYIYVDP |  | 7,64 | 8,46 | 8,94 | 7,52 | 7,79 | 8,79 |
| PGFRB_709_721 |  | P09619 | RPPSAELYSNALP |  | 6,59 | 7,04 | 7,46 | 6,49 | 6,51 | 7,35 |
| PGFRB_768_780 |  | P09619 | SSNYMAPYDNYVP |  | 5,99 | 6,56 | 6,79 | 5,76 | 6,16 | 6,70 |
| PGFRB_771_783 |  | P09619 | YMAPYDNYVPSAP |  | 5,40 | 6,32 | 6,47 | 5,56 | 6,13 | 6,39 |
| PLCG1_764_776 |  | P19174 | IGTAEPDYGALYE |  | 8,63 | 9,35 | 9,99 | 8,31 | 8,47 | 9,60 |
| PP2AB_297_309 |  | P62714 | EPHVTRRTPDYFL |  | 5,80 | 6,18 | 6,84 | 5,64 | 5,94 | 6,67 |
| PRGR_786_798 |  | P06401 | EQRMKESSFYSLC |  | 5,98 | 6,65 | 7,15 | 6,28 | 6,53 | 7,25 |
| PRRX2_202_214 |  | Q99811 | WTASSPYSTVPPY |  | 6,71 | 7,34 | 7,83 | 6,90 | 7,06 | 7,88 |
| RAF1_332_344 |  | P04049 | PRGQRDSSYYWEI |  | 7,78 | 8,29 | 9,06 | 7,85 | 7,96 | 8,89 |
| RASA1_453_465 |  | P20936 | TVDGKEIYNTIRR |  | 8,47 | 9,06 | 9,72 | 8,40 | 8,52 | 9,54 |
| RB_804_816 |  | P06400 | IYISPLKSPYKIS |  | 6,01 | 6,46 | 6,40 | 5,59 | 5,93 | 5,92 |
| RET_1022_1034 |  | P07949 | TPSDSLIYDDGLS |  | 7,30 | 8,05 | 8,56 | 7,11 | 7,30 | 8,28 |
| RON_1346_1358 |  | Q04912 | SALLGDHYVQLPA |  | 5,64 | 6,57 | 6,80 | 5,76 | 5,98 | 6,81 |
| RON_1353_1365 |  | Q04912 | YVQLPATYMNLGP |  | 5,81 | 6,35 | 7,17 | 5,58 | 5,91 | 6,86 |
| SRC8_CHICK_476_488 | | Q01406 | EYEPETVYEVAGA |  | 9,16 | 10,01 | 10,68 | 8,88 | 9,09 | 10,26 |
| SRC8_CHICK_492_504 | | Q01406 | YQAEENTYDEYEN |  | 8,34 | 9,26 | 9,80 | 8,07 | 8,42 | 9,49 |
| STAT4_714_726 |  | Q14765 | PSDLLPMSPSVYA |  | 4,81 | 5,26 | 5,96 | 4,71 | 5,13 | 5,62 |
| TEC_512_524 |  | P42680 | RYFLDDQYTSSSG |  | 5,92 | 6,70 | 7,19 | 6,06 | 6,27 | 6,78 |
| TYRO3_679_691 |  | Q06418 | KIYSGDYYRQGCA |  | 7,04 | 7,22 | 8,07 | 7,36 | 7,39 | 8,27 |
| VGFR1_1040_1052 | | P17948 | DFGLARDIYKNPD |  | 4,86 | 5,29 | 5,29 | 5,03 | 5,19 | 6,12 |
| VGFR1_1326_1338 | | P17948 | DYNSVVLYSTPPI |  | 6,48 | 7,13 | 7,67 | 6,37 | 6,62 | 7,39 |
| VGFR2_1046_1058 | | P35968 | DFGLARDIYKDPD |  | 4,72 | 5,20 | 5,64 | 4,61 | 5,12 | 6,03 |
| VGFR2_1052_1064 | | P35968 | DIYKDPDYVRKGD |  | 5,47 | 6,23 | 6,36 | 5,61 | 5,98 | 6,57 |
| VGFR2_1168_1180 | | P35968 | AQQDGKDYIVLPI |  | 5,02 | 6,07 | 6,35 | 4,86 | 5,62 | 6,18 |
| VGFR2_989_1001 |  | P35968 | EEAPEDLYKDFLT |  | 7,24 | 8,09 | 8,58 | 6,95 | 7,31 | 8,32 |
| VGFR3_1061_1073 | | P35916 | DIYKDPDYVRKGS |  | 5,63 | 6,27 | 6,17 | 5,36 | 5,63 | 6,54 |
| ZAP70_485_497 |  | P43403 | ALGADDSYYTARS |  | 5,95 | 6,98 | 7,42 | 5,96 | 6,40 | 6,98 |
| ZBT16_621_633 |  | Q05516 | LRTHNGASPYQCT |  | 6,18 | 6,36 | 6,84 | 6,46 | 6,62 | 7,45 |
